# Advancing Environmental Justice in the Community Using Charrette: A Case Study in Boston Chinatown

**DOI:** 10.1089/env.2022.0001

**Published:** 2023-11-30

**Authors:** Noelle C. Dimitri, Shir Lerman Ginzburg, Sharon Ron, Daphne Xu, Sophia Angali England, Lydia Lowe, Pilar Botana, Cristina Araujo Brinkerhoff, Samiya Haque, Doug Brugge, Linda Sprague Martinez

**Affiliations:** Dr. Noelle C. Dimitri is Assistant Professor at School of Social Work, Simmons University, Boston, Massachusetts, USA, and was previously a Doctoral Candidate at Boston University School of Social Work, Boston, Massachusetts, USA at the time this research was conducted.; Dr. Shir Lerman Ginzburg is Assistant Professor of Public Health at School of Arts and Sciences, MCPHS University, Boston, Massachusetts, USA. She was a postdoctoral fellow in Public Health at University of Connecticut School of Medicine, Farmington, Connecticut, USA.; Sharon Ron is Senior Public Health and Regional Planner at Metropolitan Area Planning Council, Boston, Massachusetts, USA.; Daphne Xu is Cultural Placekeeping Consultant at the Chinatown Community Land Trust, Boston, Massachusetts, USA.; Sophia Angali England is a student at Boston University, College of Arts and Sciences, Boston, Massachusetts, USA.; Lydia Lowe is Executive Director of the Chinatown Community Land Trust, Boston, Massachusetts, USA.; Pilar Botana is a Doctoral student in the Department of Environmental Health, Boston University School of Public Health, Boston, Massachusetts, USA.; Cristina Araujo Brinkerhoff is a Doctoral Candidate at the Boston University School of Social Work, Boston, Massachusetts, USA.; Samiya Haque is a Boston University graduate student, Boston, Massachusetts, USA.; Dr. Doug Brugge is a Professor in and chair of the Department of Public Health Sciences, University of Connecticut School of Medicine, Farmington, Connecticut, USA.; Dr. Linda Sprague Martinez is an Associate Professor and former chair of the Macro Social Work Practice Department at Boston University School of Social Work, Boston, Massachusetts, USA.

**Keywords:** traffic-related air pollution, charrette, community engagement

## Abstract

**Background:**

Community research partners in Boston Chinatown implemented a planning charrette as a part of a community-based participatory study focused on near highway research and public health action to mitigate traffic-related air pollution (TRAP). Charrettes are intensive workshops for solution-oriented design and planning used to bring together diverse stakeholders to address complex environmental health concerns.

**Methods:**

The planning charrette included three phases: (1) community meetings and resident interviews, (2) a planning charrette to address community health concerns and air pollution within larger community wellness goals, and (3) development of a Master Planning document with policy, project, and practice recommendations to guide future community advocacy.

**Outcomes:**

Intergenerational residents, community leaders, planners, researchers, and volunteers (*N* = 90) joined a day-long planning charrette to inform the Chinatown Master Plan. Workshops were informed by resident interviews focused on finding solutions to three resident identified priorities: Healthy Housing, Healthy Mobility, and Healthy Public Realm. Air pollution mitigation strategies were embedded in discussions around each priority area.

**Discussion:**

The charrette provided an opportunity for community stakeholders to voice concerns about TRAP as part of a new framework focused on health and wellness. Concerns about pedestrian safety, housing access, and expansion of green and recreational spaces were highlighted by participants as important areas for further development.

**Conclusions:**

Boston Chinatown residents reaffirmed their investment in the community by highlighting concerns about TRAP within the context of other health-related concerns. Charrettes offer a vehicle to advance environmental justice in communities through collective problem-solving and decision making.

## INTRODUCTION

Boston Chinatown is nestled in the heart of Boston's downtown commercial district, and as such, the streets are some of the most congested in the city.^1^ Heavy traffic on Chinatown streets coupled with its proximity to major highways, Interstate 93 (I-93) and the Massachusetts Turnpike (I-90), contribute to high levels of air pollution in the community.^2^ Recent reports indicate Chinatown has some of the highest levels of traffic-related air pollution (TRAP) in the Northeast region, posing significant health risks for residents.^3^

In 2007, community leaders from Boston Chinatown joined with Tufts University and community leaders from the Somerville Transportation Equity Partnership to conduct research on the health effects of near roadway pollution. Together, they formed the Community Assessment of Freeway Exposure and Health (CAFEH) community research partnership and have spent the past 14 years studying and developing interventions for TRAP.^4^ The partnership has since grown to involve community leaders and researchers from multiple organizations within Chinatown (see website link for a complete list^5^), including the Boston Chinatown Neighborhood Center, which provides a range of education, childcare, workforce, and community engagement opportunities for residents of all ages and the Chinatown Community Land Trust (CCLT), a coalition of grassroots community activists focused on the needs of working class families and the stewardship of land and monitoring of housing development within Chinatown.^6^

Furthermore, the Chinese Progressive Association and Chinatown Resident Association work in collaboration with these organizations to support a range of community initiatives focused on resident empowerment and well-being. Partners outside of Chinatown include six academic institutions, and a range of organizations, including the Metropolitan Area Planning Council (MAPC), who provides public health planning and needs assessment focused on improving the health of communities.^7^

In 2016, the CAFEH partnership was funded by the National Institute of Environmental Health Sciences to conduct a near highway research to action (R2A) study, which involved developing protective responses to mitigate ultrafine particles at the community level. This study employed community planning charrettes to generate recommendations for a health lens analysis (HLA).^8^^,^^9^ HLA is a methodology originating in South Australia used to promote collaboration among diverse community stakeholders such as residents, public health practitioners, and government staff involved in projects focused on health and well-being.^10^^,^^11^^,^^12^

Mitigating TRAP through public health action requires developing a deep understanding of community health and development priorities, which allows planners to center community concerns as they frame approaches to mitigation in contrast with imposing interventions that are not well aligned.^13^ In 2019, CAFEH partners in Chinatown conducted a community planning charrette as part of an effort to incorporate TRAP mitigation strategies in local planning efforts through the HLA process. A charrette is a data-informed planning process, which originated from the field of architectural studies.^14^^,^^15^ Charrette planning engages diverse stakeholders in community change initiatives by combining knowledge generated from diverse perspectives to produce novel and multifaceted solutions.^16^ It centers community priorities and can aid in the translation of complex research into public health action.^17^^,^^18^

The charrette process brought together public health practitioners and Chinatown leaders and residents, who were embedded in the Chinatown community, to focus on community priorities. In contrast to previous charrettes^19^ that were organized and informed primarily by university-based researchers with the goal of prompting practical real-world action to increase awareness about air pollution, this event was entirely developed by community partners in collaboration with their networks in the broader community dedicated to advancing community development and well-being, through efforts to stop displacement and by addressing everyday needs and concerns related to health, housing, education, and economic development. The partnership among these organizations predated the grant and has developed over time through organizing and social justice efforts.

Consequently, concerns about air pollution were nested in the larger community context focusing broadly on the community concerns and conditions in Chinatown. While the charrette stimulated ideas for TRAP mitigation, this focus was embedded in the larger framework of Chinatown advancing its community planning goals. In addition, the charrette was a bilingual event, with many pieces of the presentation and discussion designed and led by the community and originating in Cantonese, the dominant language in the community, and then interpreted for English-speaking participants. This approach was necessary to meaningfully engage diverse community stakeholders in planning related to community health and well-being and helped foster solution-oriented conversations.^20^

The primary goal of this article is to describe the community-driven charrette processes and outcomes in an effort to provide useful information for community planners and public health practitioners seeking to engage residents in low income, immigrant, and predominantly People of Color environmental justice (EJ) communities in health improvement efforts. The results of the planning process are presented and discussed in the context of the literature.

## BACKGROUND

The CAFEH partnership is a multicommunity agency and university consortium examining a broad range TRAP-related issues.^21^ TRAP includes the complex mixture of gaseous and particulate pollutants present in tailpipe and non-tailpipe emissions from vehicles and is associated with adverse health outcomes.^22^^,^^23^ Ultrafine particulate matter, <0.1 μm in diameter (for comparison, the thickness of human hair is ∼70 μm), is of particular concern and is associated with cardiovascular health risks including increased risk of heart attack or stroke.^24^

Because ultrafine particles are both odorless and colorless, they are often overlooked by community-based health improvement efforts. Simultaneously, communities are contending with many other health and social issues, including the lack of affordable housing.^25^ This poses challenges for efforts to address TRAP. Consequently, the CAFEH team explored ways to situate a focus on TRAP in the context of broader health and development efforts. As such, the charrette focused on how to create a healthy stable Chinatown.^26^^,^^27^

### Boston Chinatown

Chinatown is a vibrant historic neighborhood located in densely populated downtown Boston. Chinatown residents have been organizing for more than half a century to protect the neighborhood from gentrification and to advance health well-being.^28^^,^^29^ Liu situates the struggle between residents and developers in Boston Chinatown within a larger pattern of tensions between poor and working class residents and more monied developers in which residents must resist “urban production strategies” (p. 3).^30^ In Boston, a family earning <$30,000 is generally classified as being extremely low income.^31^ As of 2016, about 51.8% of all households in Chinatown are considered very low income, highlighting the tensions between residents and developers.^32^

Chinatown weathered substantial changes associated with urban renewal after expansion and construction of the highways that run adjacent and through the community as well as the expansion of Tufts Medical Center and Tufts University Medical School, which combined to result in the demolition of hundreds of housing units.^33^ More recently the neighborhood has witnessed the expansion of high-rises due to the reinvigoration of Boston's downtown. Community leaders have resisted both the development challenges and increased traffic accompanying each new phase.^34^

The completion of I-93 construction through Chinatown in the late 1950s substantially changed the community's physical landscape.^35^ The historic Chinese Merchants Association building, a central landmark, was cut in half and many other homes were razed.^36^ Subsequently, between 1962 and 1965, hundreds more Chinatown residents were displaced before immigration law was liberalized in 1965. These actions prompted a major increase in the Chinese American population.^37^ Currently, I-93 and I-90 combined carry about 300,000 vehicle trips per day through Chinatown.^38^

As a result of this congestion, Chinatown is disproportionately impacted by high TRAP levels.^39^ Research conducted by the CAFEH team identified an annual average concentration of 26,000 ultrafine particles/cm^3^ in Chinatown.^40^ This finding was concerning to both researchers and community partners, particularly since TRAP can deeply penetrate into the body and negatively impact organ function and health.^41^ As such, both groups began to think more critically about active responses to TRAP in the context of existing community organizing and planning.

TRAP disproportionately impacts EJ communities, including low-income and minoritized populations who live in proximity to high traffic roadways.^42^^,^^43^^,^^44^

In Massachusetts, EJ communities include neighborhoods meeting one or more of the following criteria: (1) the annual median household income is not >65% of the statewide annual median household income ($81,215 in 2019^45^); (2) minorities comprise 40% or more of the population; (3) 25% or more of households lack English language proficiency; or (4) minorities comprise 25% or more of the population and the annual median household income of the municipality in which the neighborhood is located does not exceed 150% of the statewide annual median household income; or (B) a geographic portion of a neighborhood designated by the Secretary of Environmental Affairs as an EJ population in accordance with law.^46^

Table 1 includes the demographic indicators from the Environmental Protection Agency EJ screening tool for Chinatown where there are higher percentiles of minority, low income, and linguistically isolated individuals.^47^ Chinatown is implicitly an EJ community and although the charrette did not frame its discussion that way explicitly, social justice, equity, and the quality of life of Chinatown residents were at the center of this project.

**Table 1.** Percentile Ranking for Chinatown Compared with All Block Groups in United States for Environmental Justice Indexes, Environmental Indicators, and Demographic Indicators from Environmental Protection Agency Environmental Justice Screen Tool

EJ indexes	Chinatown
EJ index for particulate matter (PM 2.5)	78
EJ index for NATA diesel PM	93
EJ index for NATA air toxics cancer risk	83
EJ index for NATA respiratory hazard index	84
EJ index for traffic proximity and volume	99
Demographic indicators	
Minority population	89
Low-income population	80
Linguistically isolated population	92
Population with less than high school education	98
Population under age 5	93
Population over age 64	31

From www.epa.gov/ejscreen.

EJ, environmental justice; NATA, The National-Scale Air Toxics Assessment; PM, particulate matter.

### Charrette planning

Charrette planning involves three key phases: (1) Research, Education, and Preparation; (2) the Charrette; and (3) Plan Implementation.^48^ Charrette preparation involves the collection of data to inform the planning process and is followed by a series of critical discussions each informing the next and leading to the development of a plan, with the intent that it be implemented.^49^ The charrette ideally involves 25–50 people organized into smaller groups.^50^ Charrettes adopt many illustrative strategies to develop a plan, allowing participants to visualize and conceptualize the information being generated.^51^

More recently, charrette planning has been adopted by the public health sector to advance EJ.^52^ For example, charrette was used in community planning and redesign efforts in the Mississippi Gulf Coast region in response to Hurricane Katrina.^53^ Furthermore, the CAFEH study team previously used charrettes as a tool for inspiring changes that would mitigate TRAP and address environmental injustice in Boston Chinatown.^54^

In contrast to the 2019 charrette described in this study, the earlier charrette focused on the construction of a near-highway school with an emphasis on the heating, ventilation, and air conditioning system in addition to site planning and generated community interest in advocating for the Department of Transportation to build a deck over I-93 and I-90 intersecting the Chinatown area. Although the deck was not built, likely because it is extremely expensive, subsequent to this charrette plans for the school were developed that added protective features to the air handling system.^55^

## MATERIALS AND METHODS

The evaluation component of the near highway R2A study was deemed exempt by the Boston University Institutional Review Board (protocol no. 4434X). Planning charrettes were conducted as part of the action phases of the study. The methods associated with the first two phases of the planning charrette are described in detail.

### Phase 1: Research, education, and charrette preparation

Beginning in Fall 2018, members of the research team led by Chinatown research partners engaged Chinatown residents in a series of community meetings to share their visions for a healthy Chinatown. The MAPC, the CCLT, and the Chinatown Residents Association (CRA) hosted a group of graduate students with previous training in basic interviewing skills, who conducted resident interviews in Cantonese (with interpretation) and attended CRA Steering Committee and CCLT Board Meetings. The student team provided background on prior Master Plans in Chinatown, detailed reports in English and Cantonese highlighting community development priorities and opportunities for expanding community health and stability, and facilitated conversations around the question: “How can we plan for a stable and healthy Chinatown?”

The students identified six themes focusing on public health concerns: housing, public realm, air quality, climate change, pedestrian fatalities, and open space. Next, they reviewed existing public data for these six categories and added this information to the analysis culminating in a final report summarizing their findings.^56^ TRAP was framed from the start as one of many possible concerns in Chinatown. Throughout this process the research team reflected on the ways in which TRAP intersected with the summary findings. For example, although TRAP is explicitly implicated in concerns related to air quality, it also intersects with housing and public realm as these are two areas in which TRAP exposure is a possibility. Increasing developer and planner awareness of TRAP can result in the incorporation of protective measures in community development efforts.^57^^,^^58^

In February 2019, a summary of the final report was shared with the Chinatown Master Plan Implementation Committee (MPIC), a group of Chinatown residents and representatives from community-based organizations, who provided feedback on identified health and wellness focus areas. They also expanded the list of priority areas to include community and economic development. MPIC assumed leadership over expanding the earlier student research to amplify the health and wellness of Chinatown residents captured in PLAN: Downtown,^59^ a framework developed by the Boston Planning and Development Agency (BPDA) for both preserving and enhancing the growth of Downtown Boston, including Chinatown, as a place for all to access and enjoy.

In this role, MPIC advised on format and content, and specific members were recruited to be topic area experts. The project team decided to host a 1-day community charrette focused on developing design or planning solutions, generating recommendations for PLAN: Downtown^60^ that would increase positive and minimize negative public health effects, including, but not limited to, air pollution exposure.

During the Spring and Summer of 2019, the project team conducted conversations with community organizations within and outside Chinatown (see website link for a complete list^61^) and collected additional data and stories around health priorities for the charrette. They reached out to the BPDA for additional data identified in PLAN: Downtown documents, MPIC volunteers translated these data into a series of maps and other visualizations and worked with MAPC and CCLT to organize the day, conduct outreach to participants, identify and hire translators, and recruit and orient volunteers.

### Phase 2: Charrette

#### Recruitment

Participants were recruited using a community organizing strategy that involved block and building captains engaging residents. In addition, city and staff officials, city councilors, along with a range of community agencies and their constituents, resident groups, business groups, traditional associations, and institutions with ties to Chinatown^62^ were invited to participate. Additional volunteers, including facilitators, interpreters, notetakers, community experts, technical experts (primarily researchers from CAFEH), and photographers/videographers, were recruited to help run the charrette and contribute their knowledge to discussions. A total of 90 participants took part in the design charrette.

#### Procedures

**FIG. 1.**
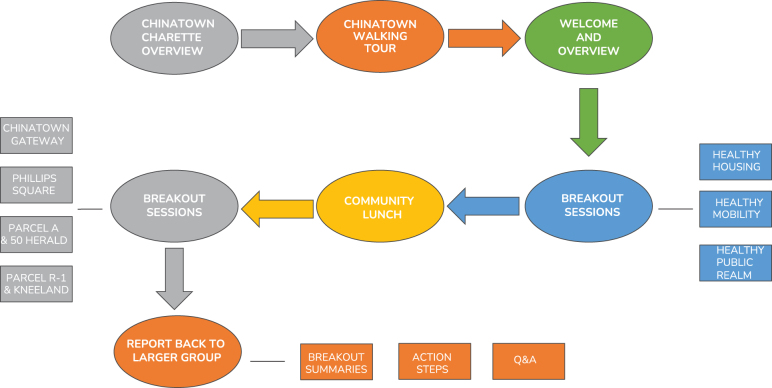
Chinatown Charrette overview.

The full-day design workshop was held in July 2019 at the Josiah Quincy Upper School in the Chinatown neighborhood, a location that was familiar and accessible to participants. An overview of the day's events is provided in Figure 1.

**FIG. 2.**
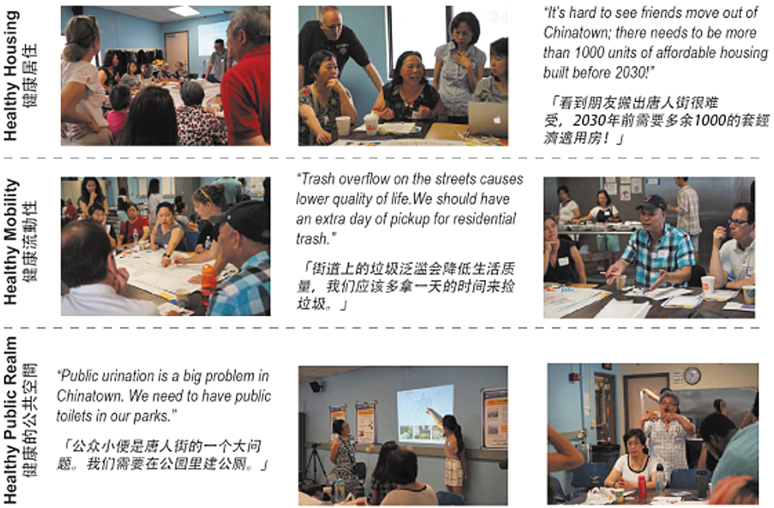
Morning breakout sessions; Graphic credit: Daphne Xu.

The workshop began with a CRA-led neighborhood tour. Next, participants joined one of three breakout sessions (Fig. 2) focused on collective problem-solving and amplifying resident voices with the goal of identifying solutions to residents' most pressing health concerns: Healthy Housing, Healthy Mobility, or Healthy Public Realm. Participant teams were provided with a document that included a variety of urban/housing design strategies sorted by topics identified by the community. The document included simple information and visuals to help navigate the design process and bilingual staff provided language translation for participants who needed it.

Facilitators for each session generated a series of questions (Supplementary Appendix SA1) to encourage participant engagement and discussion. Participants in the Healthy Housing breakout mapped out planned expansion of affordable housing in the neighborhood, the Healthy Mobility session addressed pedestrian safety and access and the Healthy Public Realm session focused on improving green and open spaces in Chinatown. Representatives from each breakout were asked to report back central themes of discussion to the larger group. TRAP emerged as one of but not the only predominant issue during the charrette. Air pollution was one of the many concerns that emerged, particularly in the afternoon sessions.

**FIG. 3.**
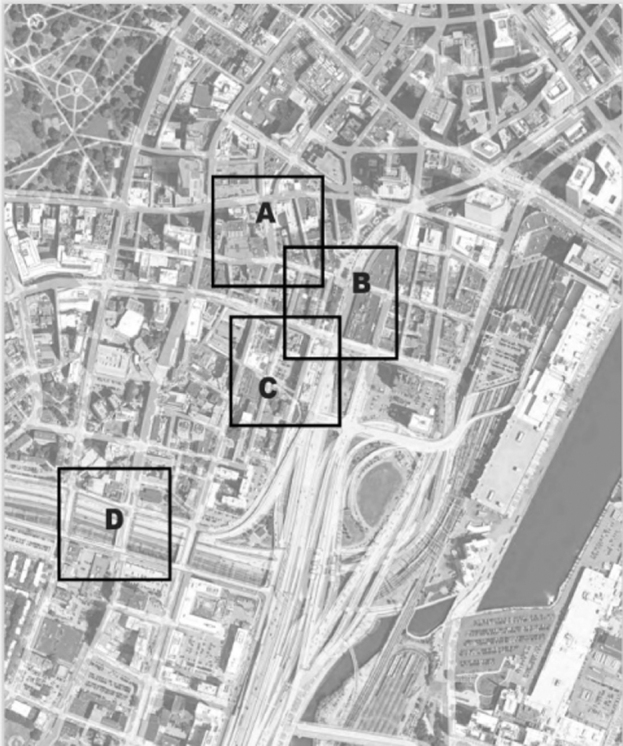
Map of opportunity area A—Phillips Square B—Chinatown Gateway C—Parcel R-1 and Kneeland D—Parcel A and 50 Herald; Source: Chinatown Master Plan. https://chinatownclt.org/chinatown-master-plan-2020

After lunch, participants joined one of four afternoon sessions addressing specific areas of opportunity, with locations marked in Figure 3, for community health intervention identified in advance by the MPIC, including the Chinatown Gateway, Phillips Square, Parcel A and 50 Herald, and Parcel R-1 and Kneeland Street. Across all these groups, participants considered strategies to mitigate air pollution within larger community health/wellness goals (Table 2).

**Table 2.** Chinatown Charrette Opportunity Areas

Opportunity area	Description	Future vision
Chinatown gateway (B)	Traditional gate marking the entrance to Chinatown	Create a welcoming, accessible, safe, and attractive space for open space users and pedestrians
Phillips square (A)	Underutilized, triangular-shaped plaza	Create an accessible and welcoming avenue to the community and serve as another “gateway” into Chinatown
Parcel A and 50 Herald (D)	Two sites earmarked for affordable housing located in proximity to I-90. (Parcel A was previously identified as a housing site and is now part of the Josiah Quincy Upper School redesign plan, whereas 50 Herald has remained a community-owned housing site)	Connect the Chinatown community over I-90; Explore affordable housing on top of Josiah Quincy Upper School on Parcel A

*Source:* Chinatown 2020 Community Vision and Strategy (2020).

### Phase 3: Plan implementation

MPIC partnered with CCLT and MAPC planners to translate the charrette findings into a Master Planning document with policy, project, and practice recommendations that will help inform community advocacy for the next 10 years. MPIC continues to meet monthly to move the Master Plan forward and organize responses to development and City initiatives.

### Outcomes

The Chinatown charrette provided an opportunity to engage participants in discussion and visual mapping around specific target areas for improving the health and quality of life for Chinatown residents. The secondary benefit of the main outcomes was that the charrette served as a helpful vehicle to explore broad community concerns about noise and air pollution. We identified three key areas described in more detail as follows, encompassing residents' concerns.

#### Healthy housing

Residents voiced concern about not being able to afford to stay in the neighborhood and emphasized the importance of preserving the integrity of the community and maintaining affordable housing, particularly for lower income residents, families, and members of the workforce. One resident shared a reflection capturing the group's sentiments: “It's hard to see friends move out of Chinatown; there needs to be more than 1000 units of affordable housing built before 2030!”^63^

Participants argued the City of Boston needed to create a minimum of 1000 housing units by 2030, a goal proposed by the Chinatown Master Plan Committee to counteract the expansion of luxury housing in the neighborhood and wanted to think beyond new developments to consider preservation of current housing. Existing and future prospective housing sites were also mapped out by participants. The development of new units creates opportunity need for TRAP mitigation efforts, such as incorporating home air filtration, especially next to the highway.

#### Healthy mobility

**FIG. 4.**
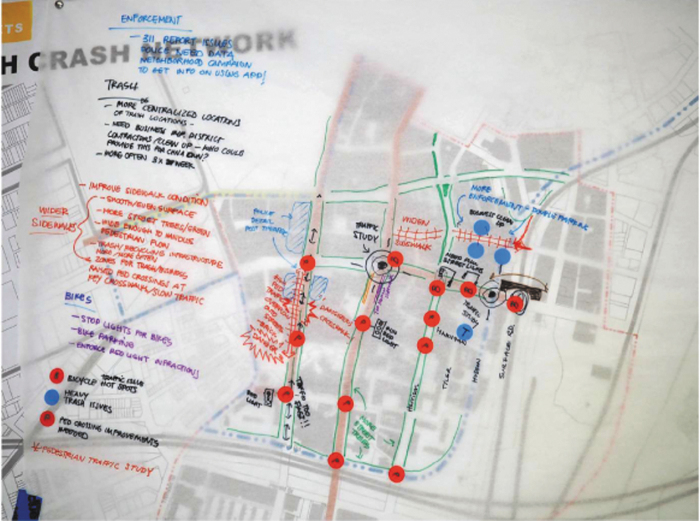
Healthy mobility map created by participants of Healthy Chinatown Design Workshop.

Participants discussed ways to increase pedestrian safety in the neighborhood's streets and marked problem intersections on a map (Fig. 4). A range of solutions were proposed including improving sidewalks using materials other than brick to minimize tripping, prioritizing pedestrians at intersections by making certain streets one-way or pedestrian-only and reducing traffic by increasing the enforcement of cycling traffic laws and designated bike traffic lights. Limiting traffic in pedestrian areas can also reduce TRAP exposure as residents navigate the community on foot.^64^ Similarly, vegetation along pedestrian pathways may help to reduce TRAP.^65^^,^^66^

Furthermore, participants suggested building a pedestrian bridge above a dangerous intersection with a highway onramp. Along with safety, participants voiced frustration with the amounts of trash littering the streets: “Trash overflow on the streets causes lower quality of life.” Trash was connected with rodent infestation, and general uncleanliness and experienced as a stressful daily obstacle for many in the group who argued for frequent garbage pickup in more congested streets and more consistent City response to complaints. Participants also proposed design changes, including creating a protected central dumping location, and widening sidewalks allowing pedestrians to more easily navigate around trash.

#### Healthy public realm

**FIG. 5.**
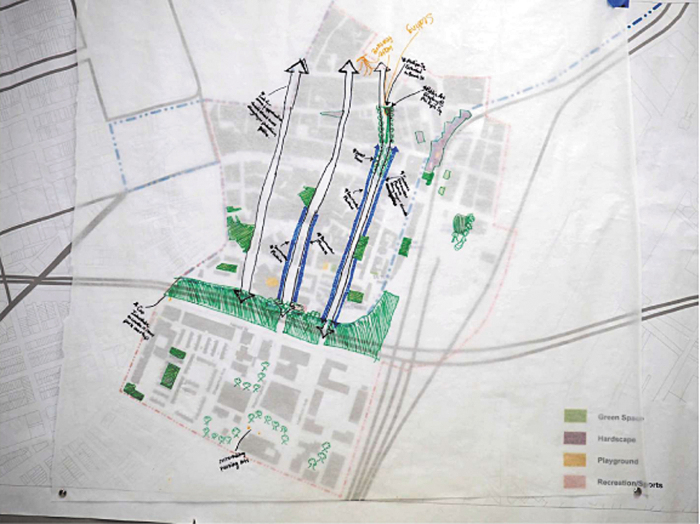
Healthy public realm map created by participants of Healthy Chinatown Design Workshop.

Residents across different generations talked about improving existing open spaces in the neighborhood by expanding public restrooms and adding water features along with vegetated barriers. The latter was seen as a means to reduce air pollution and create more visually appealing spaces (Fig. 5). Residents wondered about covering the highway and making a park deck, a structure placed over a highway, to improve community connectedness by stitching together the neighborhoods on both sides of highway and reducing resident exposure to TRAP. Residents also advocated for more greenery in their community by adding street trees and rooftop gardens. Additional measures were suggested, including increased use of permeable pavement and storm water infrastructure, in areas prone to floods.

During the afternoon breakout sessions, participants discussed four specific community locations previously identified by the Chinatown Master Plan Committee as areas of opportunity for health intervention, including ways to address air pollution. The locations included Chinatown Gateway, Phillips Square, Parcel R-1 and Kneeland Street, and Parcel A and 50 Herald Street. Each breakout session is described in more detail as follows.

**FIG. 6.**
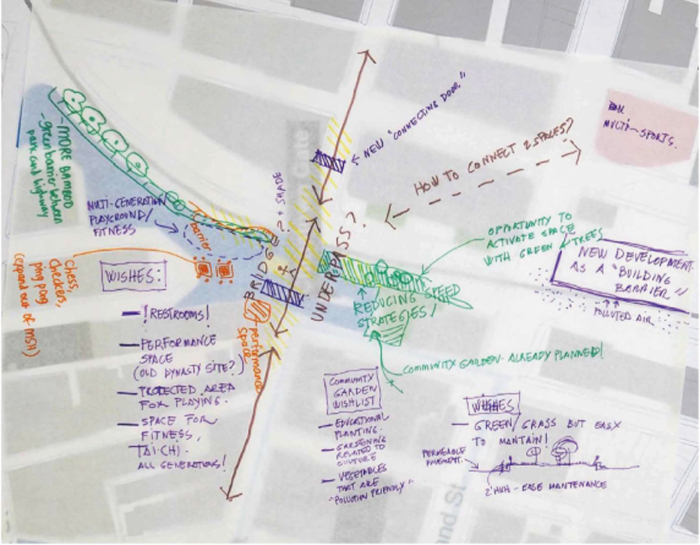
Chinatown gateway map created by participants of Healthy Chinatown Design Workshop.

First, the Chinatown Gateway area contains a vibrant archway marking the entrance to the neighborhood. Given its location in a bustling area close to Boston's downtown, participants shared concerns about traffic safety, noise, and air pollution. The group was animated while brainstorming about how to beautify the space. Participants leaned over the charrette maps to point out specific locations pedestrian access and green space could be improved (Fig. 6). Group members were eager to share their ideas about creating community gardens and expanding recreational and social spaces for residents.

Phillips Square was another space with cultural significance for participants who were passionate about creating a permanent design reflecting Chinatown's cultural history. The group envisioned the square as a place that could provide community space for all ages in the community in both warm and cold weather. Furthermore, participants discussed other ways to preserve the history and traditions of the neighborhood such as rebuilding the Ho Toy building (formerly a popular noodle company) with affordable family housing and rooftop gardening.

Housing access was something many participants raised as a pressing concern facing the Chinatown community and identified Parcel R-1 and Kneeland Street as another optimal centrally located location to expand affordable family housing. Related to this discussion, participants used the charrette maps to mark two specific traffic intersections in this vicinity needing improved traffic signaling and pedestrian crosswalks.

**FIG. 7.**
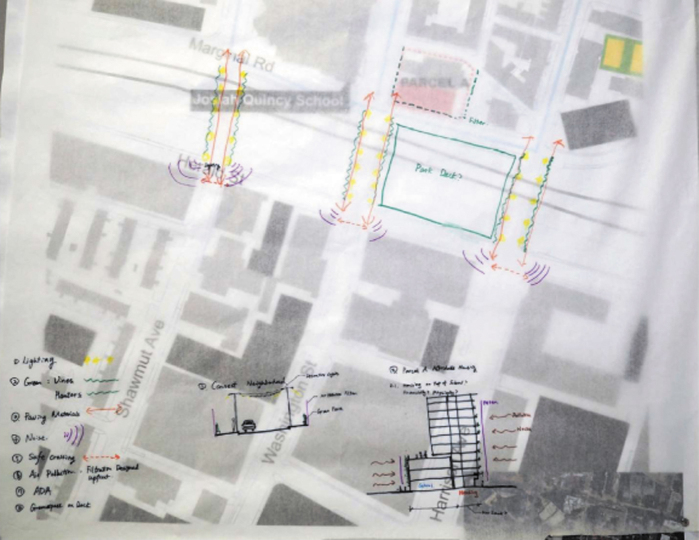
Parcel A and 50 Herald Map created by participants of Healthy Chinatown Design Workshop.

Finally, participants advocated for other priority community concerns impacting their daily quality of life such as increased rental prices, the loss of a neighborhood grocery store, air pollution, noise pollution^67^ and mitigation, and pedestrian safety and sidewalk accessibility. Overall, the pollution and noise resulting from high traffic volume in the neighborhood encroached on residents' well-being and social engagement. To mitigate these inter-related concerns, the group identified Parcel A and 50 Herald Street as another key area for redevelopment (Fig. 7). Participants suggested using lighting and landscaping to improve neighborhood connectivity and reduce TRAP and air pollution resulting from proximity to the turnpike. The group also advocated for improving Americans with Disabilities Act compliance and accessibility and voiced interest in building affordable housing on top of a school located on Parcel A.

Phase three focused on implementation, including MPIC's translation of the charrette findings into a Master Planning document with policy, project, and practice recommendations that will be used to inform community advocacy for the next 10 years. MPIC continues to meet monthly to move forward the Master Plan and organize responses to development and City initiatives. In response to this process, BPDA appears to be more aware of the issue of air quality and has made connections between the CAFEH focus on TRAP and their own energy reduction work as part of PLAN: Downtown.

## DISCUSSION

Communities face pressing public health and development concerns and TRAP, an odorless colorless threat, rarely makes it onto the agenda. Although TRAP was not the only predominant issue raised during the charrette, it remains one of many concerns for the Chinatown community. The charrette was led by people who work in and reflect the communities they serve and was focused on the current real-life concerns voiced by Chinatown residents and community organizations. This equity and resident focused lens is aligned with the “just sustainability” framework, which is defined as “the need to ensure a better quality of life for all, now and into the future, in a just and equitable manner, whilst living within the limits of supporting ecosystems” (p. 5).^68^ As a community-based participatory research partnership our approach involves centering community priorities and addressing community and environmental concerns holistically.^69^

Consistent with “just sustainability”^70^ we integrated TRAP in the context of broader community health and development priorities through charrette planning. By adopting this holistic approach, we prioritized social justice, equity, and the present-day needs of Chinatown residents while also continuing to discuss TRAP implications that allowed us to imbed TRAP mitigation in the context of the broader community health and development agenda.^71^

In keeping with the literature on charrettes, diverse stakeholders came together in the Chinatown charrette and developed concrete ideas that could improve the health and quality of life of the community.^72^^,^^73^ Community members helped the project team appreciate the benefits of bringing community leaders together to address health concerns and to consider how particular public health issues fit into the local landscape.

Consistent with the literature, we found that the charrette provided an invaluable opportunity for community members to lead the discussion about what makes their community healthy and for planners to insert the role of air pollution into the conversation. In 2014, the CAFEH study team had also organized a community charrette in Chinatown, which led to a set of community recommendations for school construction plans. More specifically, collective recommendations from the charrette resulted in the incorporation of high-quality air filtration systems into the building design plans, creating vegetation barriers between the school and the highways, and repositioning the air-intake vents of the building to the rooftop to be as far removed from the pollution origin site as possible.^74^ These initiatives established a precedent for near-highway school building construction designs in the future.^75^

The Chinatown charrette highlights the power of community-driven processes to improve community health and wellness through specific action steps. The event was a success in raising collective concern about air and noise pollution. Furthermore, bringing together residents and community leaders allowed for a fruitful equity-centered discussion of barriers to wellness in the neighborhood that can improve present-day health and quality of life for all and guide future planning.

## Supplementary Material

Supplemental data

## SUPPLEMENTARY MATERIAL

Supplementary Appendix SA1

## Notes

^1^
Linda Sprague Martinez, Noelle Dimitri, Sharon Ron, Neelakshi Hudda, Wig Zamore, Lydia Lowe, Ben Echevarria, John L. Durant, Doug Brugge, and Ellin Reisner. “Two Communities, One Highway and the Fight for Clean Air: The Role of Political History in Shaping Community Engagement and Environmental Health Research Translation.” *BMC Public Health* 20 (2020): 1690.

^2^
Allison P. Patton, Jessica Perkins, Wig Zamore, Jonathan I. Levy, Doug Brugge, and John L. Durant. “Spatial and Temporal Differences in Traffic-Related Air Pollution in Three Urban Neighborhoods Near an Interstate Highway.” *Atmospheric Environment (1994)* 99 (2014): 309–321.

^3^
Linda Sprague Martinez, Ellin Reisner, Maria Campbell, and Doug Brugge. “Participatory Democracy, Community Organizing and the Community Assessment of Freeway Exposure and Health (CAFEH) Partnership.” *International Journal of Environmental Research and Public Health* 14 (2017): 149.

^4^
CAFEH Research. <https://www.cafehresearch.org/>. (Last accessed on September 21, 2021).

^5^
Ibid.

^6^
Ibid.

^7^
Ibid.

^8^
Ibid. Martinez, Dimitri, *et al*. (2020).

^9^
Sharon Ron, Noelle Dimitri, Shir Lerman Ginzburg, Ellin Reisner, Pilar Botana Martinez, Wig Zamore, Ben Echevarria, Doug Brugge, and Linda Sprague Martinez. “Health Lens Analysis: A Strategy to Engage Community in Environmental Health Research in Action.” *Sustainability* 13 (2021): 1748.

^10^
Ibid. Martinez, Dimitri, *et al*. (2020).

^11^
Ibid. Ron *et al*. (2021).

^12^
Government of South Australia, Department of Health. (2011). “Health Lens Analysis Projects.” Version 2, 2011. <https://www.sahealth.sa.gov.au/wps/wcm/connect/public+content/sa+health+internet/about+us/about+sa+health/health+in+all+policies/health+lens+analysis+projects/health+lens+analysis+projects#:~:text=The%20Health%20Lens%20Analysis%20(HLA,the%20HiAP%20concept%20into%20action.&text=Determine%20agreed%20policy%20focus>. (Last accessed on April 4, 2022).

^13^
Linda Sprague Martinez, Wig Zamore, Alex Finley, Ellin Reisner, Lydia Lowe, and Doug Brugge. “CBPR Partnerships and Near-Roadway Pollution: A Promising Strategy to Influence the Translation of Research into Practice.” *Environments* 7 (2020): 44.

^14^
Bill Lennertz, Aarin Lutzenhiser, and Tamara Failor. “An Introduction to Charrettes.” *Planning Commissioners Journal* 71 (2008): 1–3.

^15^
Andrew Mara. “Pedagogical Approaches: Using Charettes to Perform Civic Engagement in Technical Communication Classrooms and Workplaces.” *Technical Communication Quarterly* 15 (2006): 215–236.

^16^
Rob Roggema. *Design Charrette: Ways to Envision Sustainable Futures*, 4th ed. (Springer, 2014).

^17^
Ibid. Martinez, Zamore, *et al*. (2020).

^18^
Ibid. Lennertz *et al*. (2008).

^19^
Doug Brugge, Allison P. Patton, Alex Bob, Ellin Reisner, Lydia Lowe, Oliver-John M. Bright, John L. Durant, Jim Newman, and Wig Zamore. “Developing Community-Level Policy and Practice to Reduce Traffic-Related Air Pollution Exposure.” *Environmental Justice* 8 (2015): 95–104.

^20^
Ibid. Martinez, Dimitri, *et al*. (2020).

^21^
Doug Brugge, John L. Durant, Wig Zamore, Lydia Lowe, Linda S. Sprague Martinez, Sabrina Kurtz-Rossi, Misha Eliasziw, and Barry Keppard. *Near Highway Pollution: From Research to Action*. (National Institute of Environmental Health and Safety, 2016).

^22^
Kevin J. Lane, Jonathan I. Levy, Madeleine Kangsen Scammell, Allison P. Patton, John L. Durant, Mkaya Mwamburi, Wig Zamore, and Doug Brugge. “Effect of Time-Activity Adjustment on Exposure Assessment for Traffic-Related Ultrafine Particles.” *Journal of Exposure Science & Environmental Epidemiology* 25 (2015): 506–516.

^23^
Yu Li, Kevin J. Lane, Laura Corlin, Allison P. Patton, John L. Durant, Mohan Thanikachalam, Mark Woodin, Molin Wang, and Doug Brugge. “Association of Long-Term Near-Highway Exposure to Ultrafine Particles with Cardiovascular Diseases, Diabetes and Hypertension.” *International Journal of Environmental Research and Public Health* 14 (2017): 461.

^24^
Ibid. Brugge *et al*. (2015).

^25^
Ibid.

^26^
Ibid. Ron *et al*. (2021).

^27^
Doug Brugge, Sharon Ron, Ellin Reisner, Pilar Botana, Doug Leaffer, Wig Zamore, and Barry Keppard. “Noise Barriers in Somerville: A Health Lens Analysis (HLA).” *Environmental Epidemiology* 3 (2019): 44.

^28^
Ibid. Martinez, Dimitri, *et al*. (2020).

^29^
Ibid. Roggema. (2014).

^30^
Michael Chung-Ngok Liu. *“Chinatown's Neighborhood Mobilization and Urban Development in Boston*. (University of Massachusetts Boston, 1999).

^31^
Healthy Places Design Lab, Harvard University Graduate School of Design. “Chinatown 2020 Master Plan: A Health Lens Analysis.” 2018. <https://research.gsd.harvard.edu/healthy/files/2019/06/FINAL-CHINATOWN-2020-MASTER-PLAN-A-HEALTH-LENS-ANALYSIS.pdf>. (Last accessed on April 4, 2022).

^32^
Ibid. Chung-Ngok. (1999).

^33^
Ibid. Roggema. (2014).

^34^
Ibid.

^35^
Ibid.

^36^
Ibid.

^37^
Ibid.

^38^
Ibid.

^39^
Ibid. Patton *et al*. (2014).

^40^
Ibid.

^41^
Ibid. Brugge *et al*. (2015).

^42^
Ibid. Brugge *et al*. (2015).

^43^
Luke W. Cole and Sheila R. Foster. *From the Ground Up: Environmental Racism and the Rise of the Environmental Justice Movement*. (New York University Press, 2001).

^44^
Constitution of the Commonwealth of Massachusetts, Article 97. “Environmental Justice Policy of the Executive Office of Energy and Environmental Affairs.” 2021. <https://www.mass.gov/doc/environmental-justice-policy6242021-update/download>. (Last accessed on June 24, 2021).

^45^
United States Census Bureau. “Quick Facts Massachusetts.” <https://www.census.gov/quickfacts/fact/table/MA/INC110219>. (Last accessed on December 21, 2021).

^46^
Ibid. Brugge *et al*. (2015).

^47^
United States Environmental Protection Agency. “screen.” <www.epa.gov/ejscreen>. (Last accessed on September 20, 2021).

^48^
National Charrette Institute. <https://www.canr.msu.edu/nci/>. (Last accessed on June 21, 2021).

^49^
Ibid. Constitution of the Commonwealth of Massachusetts (Article 97).

^50^
Joel A. Todd and Gail Lindsey. “Planning and Conducting Integrated Design (ID) Charrettes. Whole Building Design Guide.” <https://www.wbdg.org/resources/planning-and-conducting-integrated-design-id-charrettes>. (Last accessed on December 21, 2021).

^51^
Ibid. Mara. (2006).

^52^
Jason B. Walker and Michael W. Seymour. “Utilizing the Design Charrette for Teaching Sustainability.” *International Journal of Sustainability in Higher Education* 9 (2008): 157–169.

^53^
Ibid. United States Environmental Protection Agency.

^54^
Ibid. Brugge *et al*. (2019).

^55^
Ibid.

^56^
Metropolitan Area Planning Council (MAPC). “Chinatown Master Plan 2020.” <https://www.mapc.org/resource-library/chinatown-master-plan-2020/>. (Last accessed on December 21, 2021).

^57^
Ibid. Martinez, Dimitri, *et al*. (2020).

^58^
Ibid. Roggema. (2014).

^59^
PLAN: Downtown. <www.bostonplans.org/planning/planning-initiatives/plan-downtown>. (Last accessed on December 21, 2021).

^60^
Ibid. Todd and Lindsey.

^61^
Ibid. CAFEH Research.

^62^
Ibid. National Charrette Institute.

^63^
Ibid.

^64^
Ibid. Brugge *et al*. (2019).

^65^
Ibid.

^66^
Richard Baldauf. “Roadside Vegetation Design to Improve Local, Near-Road Air Quality.” *Transportation Research. Part D, Transport and Environment* 52 (2017): 354361.

^67^
Erica D. Walker, Nina F. Lee, Madeleine K. Scammell, Arielle P. Feuer, Maria B. Power, Kevin J. Lane, Gary Adamkiewicz, and Jonathan I. Levy. “Descriptive Characterization of Sound Levels in an Environmental Justice City Before and During a Global Pandemic.” *Environmental Research* 199 (2021): 111353.

^68^
Julian Agyeman, Robert D. Bullard, and Bob Evans. *Just Sustainabilities: Development in an Unequal World*. (Taylor & Francis, 2003).

^69^
Ibid. Agyeman *et al*. (2003).

^70^
Ibid.

^71^
Ibid.

^72^
Ibid. Brugge *et al*. (2019).

^73^
Ibid. Constitution of the Commonwealth of Massachusetts (Article 97).

^74^
Ibid. Brugge *et al*. (2019).

^75^
Ibid.

