# Supplementary material for: Advancing Environmental Justice in the Community Using Charrette: A Case Study in Boston Chinatown
Source: Environ Justice. 2023 Nov 30;16(6):461–72. doi: 10.1089/env.2022.0001 (PMC10704576; doi:10.1089/env.2022.0001)
Supplement: Supplemental data [file Suppl_AppendixSA1.docx]

Appendix: Chinatown Charrette Breakout Session Discussion Prompts

Morning Breakout Sessions

1. Healthy Housing
2. How might we preserve the culture of Chinatown as a working-class immigrant neighborhood through the affordability of housing?
3. How do we preserve current affordable housing and keep current Chinatown residents in their homes and communities?
4. How might we reach the goal of 1000 new affordable housing units in Chinatown by 2025 set out in 2014?
5. What should Chinatown’s affordable housing goals be for 2030?
6. How might affordable housing units be designed to suit the needs of varied resident groups?
7. What are the different living needs of these varied resident groups?
8. Who should the new affordable housing be designed for?
9. Healthy Mobility
10. How might we ensure pedestrian safety on Chinatown’s streets for varied Chinatown users of all ages?
11. How might we ensure the cleanliness of Chinatown’s streets and sidewalks, especially within the Beach Street area?
12. Do we need more parking or do we want less cars on the road?
13. How might we achieve zero pedestrian injuries/deaths from traffic-related accidents?
14. What can be done to increase public safety, improve the pedestrian environment, and engage in transportation planning to address community needs ?
15. Healthy Public Realm
16. How might we create a network of green/open spaces for varied Chinatown users?
17. What steps do we take to have 27% of Chinatown under tree canopy coverage?
18. What can be done to open a permanent Chinatown Library on Parcel 12C?
19. How do different Chinatown residents use open space?
20. How can Chinatown’s open spaces be multigenerational?
21. How might we build the resilience of Chinatown through a green infrastructure strategy (to mitigate risks of flooding) ?

**List of Charette Community Agencies and Partners**

Asian Community Development Corporation

Chinatown Community Land Trust

Chinatown Main Street

Chinatown Master Plan Committee

Chinatown Resident Association, Chinese Consolidated Benevolent Association

Chinese Progressive Association

Boston City Councilor Ed Flynn

Representatives from MAPC

The Josiah Quincy Upper School

Architectural, engineering, and design firms.
